# Supplementary material for: Semigroup-theoretic analysis of supply-chain disruptions and resilience
Source: PLoS One. 2026 May 28;21(5):e0350123. doi: 10.1371/journal.pone.0350123 (PMC13218532; doi:10.1371/journal.pone.0350123)
Supplement: S1 Appendix — This appendix provides supplementary transformation data, reachability tables, and computational procedures supporting the theoretical analysis developed in the main text. The examples illustrate how disruption and recovery processes in manufacturing, agricultural, and e-commerce supply chains can be represented as finite transformation semigroups. (DOCX) [file pone.0350123.s001.docx]

Supporting information

**S1 Appendix.** **Supplementary transformation data and computational procedures.** This appendix provides supplementary transformation data, reachability tables, and computational procedures supporting the theoretical analysis developed in the main text. The examples illustrate how disruption and recovery processes in manufacturing, agricultural, and e-commerce supply chains can be represented as finite transformation semigroups.

All examples are constructed to demonstrate synchronising collapse behaviour, idempotent stabilisation, and minimal collapse-inducing generator sets consistent with the structural results established in Sections Disruptions and interventions as transformations and Reachability lattices and shock-propagation geometry.

Example transformation matrices

In each case, the system state space is finite and represents discrete levels of operational performance. Transformations correspond to disruptions or interventions that modify system configurations.

**Manufacturing supply chain.** Consider the state set <<Eqn168>> where *x*_0_ represents normal production, *x*_1_ reduced production, *x*_2_ severe backlog, and *x*_3_ system collapse. The transformation table is shown in Table 8.

Table 8. **Manufacturing transformation matrix.**

| **Transformation** | *x*_0_ | *x*_1_ | *x*_2_ | *x*_3_ |
| --- | --- | --- | --- | --- |
| *d*_port_ | *x*_2_ | *x*_2_ | *x*_3_ | *x*_3_ |
| *d*_supplier_ | *x*_1_ | *x*_2_ | *x*_2_ | *x*_3_ |
| *r*_reroute_ | *x*_0_ | *x*_0_ | *x*_1_ | *x*_3_ |

Repeated application of *d*_port_ produces a synchronising collapse state: <<Eqn169>> for all <<Eqn170>>.

**Agricultural supply chain.** Let <<Eqn171>> represent agricultural production states: normal harvest, weather disruption, distribution delay, and market shortage. The transformation matrix is shown in Table 9.

Table 9. **Agricultural transformation matrix.**

| **Transformation** | *a*_0_ | *a*_1_ | *a*_2_ | *a*_3_ |
| --- | --- | --- | --- | --- |
| *d*_weather_ | *a*_1_ | *a*_2_ | *a*_3_ | *a*_3_ |
| *d*_transport_ | *a*_2_ | *a*_2_ | *a*_3_ | *a*_3_ |
| *r*_redistribute_ | *a*_0_ | *a*_1_ | *a*_1_ | *a*_3_ |

State *a*_3_ represents systemic shortage and functions as a collapse configuration.

**E-commerce logistics network.** Let <<Eqn172>> denote logistics system states: normal delivery operations, delivery delays, warehouse congestion, and fulfilment failure. The transformation matrix is shown in Table 10.

Table 10. **E-commerce transformation matrix.**

| **Transformation** | *e*_0_ | *e*_1_ | *e*_2_ | *e*_3_ |
| --- | --- | --- | --- | --- |
| *d*_routing_ | *e*_1_ | *e*_2_ | *e*_3_ | *e*_3_ |
| *d*_warehouse_ | *e*_2_ | *e*_2_ | *e*_3_ | *e*_3_ |
| *r*_reallocate_ | *e*_0_ | *e*_1_ | *e*_1_ | *e*_3_ |

The collapse state *e*_3_ corresponds to complete fulfilment failure.

Reachability lattice tables

Reachability relations describe the configurations accessible under repeated applications of transformations.

**Manufacturing reachability.** The reachability table is shown in Table 11.

Table 11. **Manufacturing reachability table.**

| **State** | **Reachable states** |
| --- | --- |
| *x*_0_ | <<Eqn173>> |
| *x*_1_ | <<Eqn174>> |
| *x*_2_ | <<Eqn175>> |
| *x*_3_ | {*x*_3_} |

This ordering defines a finite lattice under reachability.

Minimal collapse-inducing generator sets

A generator set is minimal if it produces collapse behaviour and no proper subset produces the same effect (Table 12).

Table 12. **Minimal collapse-inducing generator sets.**

| **Sector** | **Minimal generator set** |
| --- | --- |
| Manufacturing | { *d*_port_ } |
| Agriculture | <<Eqn176>> |
| E-commerce | <<Eqn177>> |

These generator sets correspond to disruption combinations sufficient to drive the system into collapse states.

Sample computational scripts

The following pseudocode illustrates the computation of reachability sets for a finite transformation semigroup:

# Example transformation dictionary

transformations = {

    ’d_port’: {

        ’x0’: ’x2’,

        ’x1’: ’x2’,

        ’x2’: ’x3’,

        ’x3’: ’x3’

    }

}

def reachable_states(start, transform):

    visited = set()

    current = start

    while current not in visited:

        visited.add(current)

        current = transform[current]

    return visited

print(reachable_states(’x0’,

      transformations[’d_port’]))

The script computes reachable configurations under repeated application of a single disruption transformation. Similar procedures can be extended to compute reachability lattices, synchronising transformations, and minimal generator sets.
