# Supplementary material for: Semigroup-theoretic analysis of supply-chain disruptions and resilience
Source: PLoS One. 2026 May 28;21(5):e0350123. doi: 10.1371/journal.pone.0350123 (PMC13218532; doi:10.1371/journal.pone.0350123)
Supplement: Attachment — Submitted filename: Review_PlosOne_SemigroupTheoreticAnalysis.pdf [file pone.0350123.s002.pdf]

Review
Semi-group Theoretic Analysis of Supply-Chain Disruptions and Resilience
Submitted to Plos One

This is a completely theoretic paper with interesting applications of semi-group analysis to supply chains. It is interesting how authors relate the algebraic structure to disruptions and residence of supply chains in particular theoretic escenarios. However, some comments point out changes that should be made in order to be suited for publication.

- The theoretic analysis presented in the paper does complement existing literature such as network analysis and operational modeling. However, the paper is missing a review of related literature of such analyses. Authors should include in this literature review advances in network propagation models and application using data.
- Make a clearer presentation of contribution in contrast to network analysis.
- My main concern is regarding practical applicability to non-mathematicians. Despite the good theoretic presentation, authors should improve the explanation and intuition behind applied examples of supply chains. Supply chains are an important subject in economics and, as the paper currently stands, it will be hard for economists to follow and apply the algorithms proposed. This will be even harder for policy makers.
- In complement with my previous point, authors should improve presentation of the case study considering the analysis of economic situations.
- All theoretic data should be publicly available through a repository such as an Open Science Framework.

Recommendation: addressed all comments before being suited for publication.
