# Supplementary material for: Semigroup-theoretic analysis of supply-chain disruptions and resilience
Source: PLoS One. 2026 May 28;21(5):e0350123. doi: 10.1371/journal.pone.0350123 (PMC13218532; doi:10.1371/journal.pone.0350123)
Supplement: Attachment — Submitted filename: Response to Reviewer-Semigroup in Supply Chain.pdf [file pone.0350123.s004.pdf]

Response to Reviewer

Manuscript Title:
Semigroup Theoretic Analysis of Supply-Chain Disruptions and Resilience

Journal: PLOS ONE

We sincerely thank the reviewer for the careful reading of our manuscript and for the constructive suggestions provided. We have revised the manuscript accordingly and addressed each comment in detail below. All changes have been incorporated into the revised manuscript to improve clarity, practical interpretation, and reproducibility.

Reviewer Comment 1

"The theoretic analysis presented in the paper does complement existing literature such as network analysis and operational modeling. However, the paper is missing a review of related literature of such analyses. Authors should include in this literature review advances in network propagation models and application using data."

Response

We appreciate this important suggestion. In response, we have expanded the Introduction to include a discussion of recent advances in network propagation models and data-driven disruption analysis. The revised text now situates the semigroup-based framework within the broader context of supply-chain modeling approaches, including network-based and simulation-based methods.

These additions clarify how algebraic transformation models provide complementary structural insights beyond probabilistic or graph-based propagation models.

Location in Manuscript

Introduction section — literature review paragraph discussing network propagation and data-driven disruption models, with additional references included.

Reviewer Comment 2

"Make a clearer presentation of contribution in contrast to network analysis."

Response

We have revised the final paragraph of the Introduction to explicitly state the distinct contribution of the semigroup-theoretic approach relative to traditional network-based models.

The revised text now emphasizes that the proposed framework focuses on structural properties of disruption transformations, including collapse states, reachability, and stabilisation behaviour, which are not directly captured by standard network flow or simulation models.

This clarification strengthens the positioning of the work within the broader supply-chain modeling literature.

Location in Manuscript

Introduction section — concluding paragraph describing the theoretical and operational contributions of the proposed framework.

Reviewer Comment 3

"My main concern is regarding practical applicability to non-mathematicians. Despite the good theoretic presentation, authors should improve the explanation and intuition behind applied examples of supply chains. Supply chains are an important subject in economics and, as the paper currently stands, it will be hard for economists to follow and apply the algorithms proposed. This will be even harder for policy makers."

Response

We thank the reviewer for highlighting the importance of accessibility for practitioners and policy-oriented readers. To address this concern, we introduced additional explanatory text throughout the examples and case discussions to provide operational interpretation of the mathematical constructs.

Specifically, we added descriptive paragraphs explaining:

- how disruptions correspond to operational events such as port closures or logistics failures,
- how repeated disruptions lead to backlog accumulation,
- how stabilisation corresponds to restoration of operational capacity,
- how collapse states represent conditions requiring external intervention.

These additions provide intuitive explanations linking the algebraic framework to real-world supply-chain behaviour.

Location in Manuscript

Immediately before the manufacturing disruption example — new subsection titled:

Operational Interpretation

This section explains the practical meaning of collapse states and disruption transformations in manufacturing and logistics systems.

Reviewer Comment 4

"In complement with my previous point, authors should improve presentation of the case study considering the analysis of economic situations."

Response

In response to this suggestion, we strengthened the practical interpretation of the case study examples by explicitly linking the mathematical framework to observable economic and logistics disruptions.

The revised manuscript now includes:

- clearer descriptions of production and delivery interruptions,
- interpretation of collapse states as operational failure scenarios,
- examples of corrective actions such as rerouting shipments or restoring infrastructure,
- structured transformation examples representing logistics disruptions.

In addition, we included a supplementary appendix containing illustrative transformation matrices and reachability tables that demonstrate how disruptions propagate through supply-chain states.

These additions improve the economic relevance and interpretability of the examples while preserving the theoretical rigor of the model.

Location in Manuscript

Case study and example sections — expanded explanatory paragraphs.

Appendix — supplementary transformation and reachability examples.

Reviewer Comment 5

"All theoretic data should be publicly available through a repository such as an Open Science Framework."

Response

We appreciate this recommendation regarding transparency and reproducibility. In response, we have added a formal Data Availability section to the manuscript describing how the data underlying the practical illustrations can be accessed.

The revised manuscript now states that:

- theoretical examples and computational materials are available from the corresponding author upon reasonable request,
- practical disruption illustrations are based on publicly available logistics and port performance datasets.

The dataset sources are now explicitly cited in the manuscript.

To support reproducibility, we also included supplementary materials in the appendix containing:

- representative transformation matrices,
- reachability tables,
- minimal collapse-inducing generator sets,
- sample computational scripts.

These materials allow readers to reproduce the structural analyses presented in the paper.

Location in Manuscript

Data Availability section — newly added.

Appendix — supplementary transformation data and computational procedures.

Summary of Revisions

The revised manuscript now includes:

- Expanded literature review on network propagation and data-driven supply-chain analysis,
- Clearer statement of the contribution of semigroup-based modeling,

- Improved operational interpretation of mathematical examples,
- Stronger economic and logistics context for case studies,
- Formal data availability statement and supplementary materials supporting reproducibility.

We believe these revisions fully address the reviewer's concerns and significantly strengthen the clarity, applicability, and transparency of the manuscript.

We sincerely thank the reviewer for the thoughtful comments that helped improve the quality and accessibility of this work.
